# Tablet Disintegration and Dispersion under In Vivo-like Hydrodynamic Conditions

**DOI:** 10.3390/pharmaceutics14010208

**Published:** 2022-01-16

**Authors:** Jan Lenz, Frederik Fuest, Jan Henrik Finke, Heike Bunjes, Arno Kwade, Michael Juhnke

**Affiliations:** 1 Technical Research & Development, Novartis Pharma AG, Fabrikstrasse 2, CH-4056 Basel, Switzerland; michael.juhnke@novartis.com; 2 Research & Development, LaVision GmbH, Anna-Vandenhoeck-Ring 19, D-37081 Göttingen, Germany; ffuest@lavision.de; 3 Institut für Partikeltechnik, Technische Universität Braunschweig, Volkmaroder Strasse 5, D-38104 Braunschweig, Germany; jan.finke@tu-braunschweig.de (J.H.F.); a.kwade@tu-braunschweig.de (A.K.); 4 Zentrum für Pharmaverfahrenstechnik—PVZ, Technische Universität Braunschweig, Franz-Liszt-Strasse 35a, D-38106 Braunschweig, Germany; heike.bunjes@tu-braunschweig.de; 5 Institut für Pharmazeutische Technologie und Biopharmazie, Technische Universität Braunschweig, Mendelssohnstrasse 1, D-38106 Braunschweig, Germany

**Keywords:** tablet disintegration, tablet performance, hydrodynamics, in vivo conditions, pharmaceutical polymers, disintegrants, tomographic imaging

## Abstract

Disintegration and dispersion are functional properties of tablets relevant for the desired API release. The standard disintegration test (SDT) described in different pharmacopoeias provides only limited information on these complex processes. It is considered not to be comparable to the biorelevant conditions due to the frequent occurrence of high hydrodynamic forces, among other reasons. In this study, 3D tomographic laser-induced fluorescence imaging (3D Tomo-LIF) is applied to analyse tablet disintegration and dispersion. Disintegration time (DT) and time-resolved particle size distribution in close proximity to the tablet are determined in a continuously operated flow channel, adjustable to very low fluid velocities. A case study on tablets of different porosity, which are composed of pharmaceutical polymers labelled with a fluorescent dye, a filler, and disintegrants, is presented to demonstrate the functionality and precision of the novel method. DT results from 3D Tomo-LIF are compared with results from the SDT, confirming the analytical limitations of the pharmacopoeial disintegration test. Results from the 3D Tomo-LIF method proved a strong impact of fluid velocity on disintegration and dispersion. Generally, shorter DTs were determined when cross-linked sodium carboxymethly cellulose (NaCMCXL) was used as disintegrant compared to polyvinyl polypyrrolidone (PVPP). Tablets containing Kollidon VA64 were found to disintegrate by surface erosion. The novel method provides an in-depth understanding of the functional behaviour of the tablet material, composition and structural properties under in vivo-like hydrodynamic forces regarding disintegration and the temporal progress of dispersion. We consider the 3D Tomo-LIF in vitro method to be of improved biorelevance in terms of hydrodynamic conditions in the human stomach.

## 1. Introduction

Tablet disintegration and dispersion are characteristic subprocesses of the active pharmaceutical ingredient (API) release process [1,2,3,4,5]. While disintegration generally represents the break-up of the tablet into fragments, dispersion particularly describes the processes of deagglomeration or deaggregation of these fragments subsequent to disintegration, where a further size reduction into primary particles is reached [5]. The transition from disintegration to dispersion is not exactly defined. The temporal progress of tablet disintegration and dispersion as well as the size of agglomerates, aggregates and primary particles may impact the API release.

Thus, the time-resolved characterisation of both disintegration and dispersion are important for the development of tablet formulations. However, such a characterisation is mostly only referred to as disintegration testing. The standard in vitro disintegration test (SDT), which is described in the harmonised monographs of the pharmacopoeias, is widely used and has become an international standard test method [6,7,8]. In this test, up to six tablets can be tested simultaneously, where each of the tablets is placed in a cylindrical and transparent tube, and mounted on a basket in a vertical position. Separate screens with a mesh size of 2 mm are installed at the bottom plate of the basket, covering the lower end of each tube. This basket is immersed in a test liquid of 35 to 39 °C inside a beaker and alternatingly moved in a vertical direction at a constant number of cycles per minute. The disintegration time (DT) is determined for each tablet when complete disintegration is reached. In the USP, it is written that “complete disintegration is defined as that state in which any residue of the unit, except fragments of insoluble coating […], remaining on the screen of the test apparatus […], is a soft mass having no palpably firm core” [6]. The SDT is used as a quality control test in tablet manufacturing, as well as for the characterisation of the tablet performance for formulation development. The tablets are exposed to hydrodynamics due to the sinusoidal movement of the basket. An average fluid velocity of 55 mm/s and a maximum value of 85 mm/s throughout the test can be estimated according to the moving velocity of the basket [9,10]. Kindgen et al. simulated the hydrodynamic conditions in the SDT set-up and reported higher maximum fluid velocities of nearly 250 mm/s and 200 mm/s for Newtonian and non-Newtonian test media, respectively. They found a decrease in fluid velocity with increasing fluid viscosity and, therefore, different flow patterns near the tablets [10]. Besides the impact on the hydrodynamic conditions in the SDT, increasing viscosity of real and simulated gastric content increases the DT of tablets [9]. While the simplicity of the SDT may be considered advantageous, its biorelevance regarding the hydrodynamic conditions and the utilised test media is debated [11]. 

Different approaches have been made to gain insights into the in vivo hydrodynamic conditions within a human stomach. In the fasted state, the hydrodynamics in the stomach are determined by the migrating motor complex (MMC), which is a cycle of predominantly quiet flow with occasional short periods of contraction pulses, and a total duration of 90–120 min [12,13]. In the fed state, there are two main flow patterns in the gastric region, namely retropulsion and recirculation [14]. In general, retropulsion generates higher fluid velocities, but frequency and duration are lower compared to recirculation [15]. Katori et al. estimated the fluid velocities in the stomach by comparing the results of in vitro dissolution experiments carried out in a flow-through cell at different flow rates with in vivo drug release profiles and found a correlation with the in vitro experiment at 0.15 mm/s [16]. O’Grady et al. measured in vivo fluid velocities between 3 and 8 mm/s in different regions of a human stomach using high resolution mapping [17]. Boulby et al. utilised magnetic resonance imaging for in vivo investigations into the gastric flow and reported maximum fluid velocities for retropulsion between 36 and 52 mm/s. In contrast, Hausken et al. found maximum fluid velocities for retropulsion of 200 mm/s using duplex sonography [15]. However, these high velocities must be considered as local pulse peaks with a duration of only a few seconds and a frequency of 1–3 per minute [15,18,19,20]. 

Besides in vivo analytics, computer simulations were employed to understand the hydrodynamics within a human stomach. Pal et al. performed 2D simulations assuming a viscosity of the gastric content of 1 Pa∙s. They reported fluid velocities at pulse peak of 7.5 mm/s for retropulsion and of 2 mm/s for the recirculating flow [21]. Imai et al. calculated fluid velocities at pulse peak of 30 mm/s for retropulsion and average recirculation velocities < 1 mm/s using numerical simulations assuming the gastric content to be a Newtonian fluid with a viscosity of 1 Pa∙s [14]. Ferrua et al. designed a 3D computational fluid dynamic model of the stomach and investigated the effect of viscosity of the gastric content. They found fluid velocities at pulse peak for retropulsion of 76 mm/s for the lower viscosity (10^−3^ Pa∙s) and of 120 mm/s for the higher viscosity (1 Pa∙s) [22,23]. In summary, the hydrodynamic conditions in the human stomach are quite complex and are subjected to high unsteadiness and variability. Thus, an exact simulation of the in vivo hydrodynamic conditions with an in vitro test set-up is not practicable. However, the simulation of the dominating flow regime in consideration of the least favourable conditions for the processes of tablet disintegration and dispersion is a pragmatic approximation. This approximation enables the experimental investigation of the functional behaviour of tablet disintegration and dispersion under in vivo-like hydrodynamic conditions. A tablet is mainly exposed to relatively low fluid velocities of a few millimetres per second within the human stomach in the fasted state and during recirculation activity in the fed state. These are supplemented by short pulse peaks of about one order of magnitude higher fluid velocities during MMC and retropulsion activity; however, this occurs at very low frequency. A higher flexibility regarding the hydrodynamic test conditions, including the fluid velocity, was suggested to improve the comparability of in vivo and in vitro disintegration [9,10,24]. 

Kindgen et al. developed a modified device based on the SDT, which could simulate varying hydrodynamic conditions by a controllable vertical movement of the basket and allowed horizontal fluid flow towards the tablets [24]. They showed the impact of fluid velocity and shear stress on DT.

Dvořák et al. investigated tablet disintegration in a stepwise way by employing three individual analytical methods [25]. Magnetic resonance imaging was used to detect structural changes of the tablets, texture analysis to measure the disintegration kinetics and static light scattering to characterise the size of the fragments after complete disintegration. Although useful information on the disintegration process could be obtained, each analytical step was carried out separately and required an individual tablet. The results obtained from the individual analytical methods need to be related to each other for proper interpretation under consideration of complex interactions with other processes such as dissolution or swelling of excipients. 

Coutant et al., Xu et al. and Wilson et al. investigated disintegration of a single tablet in a stirred vessel by monitoring the cumulative progress of dispersion by different time-resolved analytical techniques [26,27,28]. Coutant et al. and Xu et al. utilised inline focused beam reflectance measurements to determine the chord length distribution of the disintegrated particles in the stirred vessel [26,27]. However, the chord length distribution cannot be easily converted into a particle size distribution [29]. Wilson et al. analysed the number concentration and size of disintegrated particles by online dynamic image analysis. The particle suspension was transported from the vessel to the analytical device by a peristaltic pump, which could have led to a further reduction in particle size [28]. Coutant et al. and Wilson et al. used a stirred vessel according to the USP 2 dissolution method in their experiments. The hydrodynamics in the USP 2 set-up were investigated by computational fluid dynamic simulations. It was found that the hydrodynamics, the fluid velocity in particular, in the surrounding of the tablet varied significantly within a radius of only a few millimetres, reaching from close to 0 up to 1/3 of the paddle stirrer tip speed [30]. These disintegration tests can be considered to be informative regarding the comparison with USP 2 dissolution data. However, biorelevant disintegration behaviour might be difficult to simulate.

Quodbach and Kleinebudde presented a time-resolved method to analyse the development of the cumulative particle size distribution after disintegration by modified spatial filtering velocimetry in a circulating flow with an adjusted fluid velocity of 640 mm/s [31]. The analysis provided useful information on the cumulative dispersion of disintegrated particles. 

However, the methods described besides the SDT do not involve analysis of the tablets as such and, therefore, a DT was not determined [26,27,28,31]. Further, a discrimination between particles liberating at different time points could not be made. During such a cumulative dispersion analysis, possible changes of the particle size over time, which may be caused by parallel or subsequent processes such as swelling or dissolution of excipients or APIs, respectively, have an impact on the results. The interpretation of obtained particle size trajectories might be difficult in some cases.

Mesnier et al. introduced a method to analyse both the tablet and the particles generated during disintegration [32]. They placed a tablet in a flow cell, generated a directed bottom to top flow through the cell and recorded the tablet and a fixed volume above the tablet with a single digital camera. The tablet dimensions and the size of the particles including agglomerates, aggregates and primary particles directly after disintegration in close proximity to the tablet were determined by image analysis. Superimposed particles could not be discriminated. When particles are analysed in a laminar bottom to top flow, inertial and gravitational forces act in opposite directions. Thus, the direction of the particles moving in the flow depends on which of these forces predominates. Sedimentation of the particles can be prevented by increasing the fluid velocity. However, with increasing fluid velocity the variability of the hydrodynamic test conditions decreases. Rajkumar et al. used an experimental set-up similar to Mesnier et al. and reported the need of a fluid velocity of 24 mm/s to avoid sedimentation of MCC particles in the flow channel [33]. 

Challenges in disintegration may frequently occur for formulations containing amorphous solid dispersions (ASDs). ASDs are a frequently applied formulation strategy for poorly water-soluble APIs. The APIs are dispersed in a solid amorphous carrier, which is often a pharmaceutical polymer [34]. A number of such polymers are prone to form a gelling polymer network (GPN), hindering the disintegration of tablets containing ASDs [35]. In several studies, significantly increased DTs (as determined by the SDT) were reported for tablets with a high content of specific polymers due to the formation of GPN [36,37,38]. Performing such an analysis under more in vivo-like hydrodynamic conditions would possibly lead to a further increase in the DTs. Thus, a method to analyse the disintegration and progress of the dispersion of tablets containing pharmaceutical polymers under more in vivo-like hydrodynamic conditions might be beneficial for the formulation development of tablets containing ASDs.

The aim of this study is the development of an in vitro method to investigate the disintegration of tablets and dispersion of liberated particles containing pharmaceutical polymers used for ASDs at more in vivo-like hydrodynamic conditions in order to improve biorelevance and bioprediction. The determination of tablet DT and the time-resolved dispersion analysis of the disintegrated polymer particles are performed at low fluid velocities, which covers the approximated range of biorelevant fluid velocities in the human stomach for the fasted state and for recirculation activity in the fed state. A case study was performed on tablets containing a pharmaceutical polymer, a filler and a disintegrant. Porosity, polymer type, disintegrant type and disintegrant content were systematically varied to demonstrate the sensitivity and capability of the analytical method. The results are compared with a complementary analysis of the DT using the SDT at standard (55 mm/s) and modified (9 mm/s) average fluid velocity, based on the average basket velocity.

## 2. Materials and Methods

### 2.1. Materials

In this study, two pharmaceutical polymers, which are commonly used as carriers for the manufacturing of ASDs, vinylpyrrolidone vinylacetate copolymer (Kollidon VA64, BASF, Basel, Switzerland) and aminoalkyl methacrylate copolymer (Eudragit EPO, Evonik, Essen, Germany) were investigated in a formulation with fillers and disintegrants. Microcrystalline cellulose (MCC, Vivapur^®^102, JRS Pharma, Rosenberg, Germany), a hydrophilic and water-insoluble excipient with plastic deformation behaviour, was used as filler [39]. Cross-linked sodium carboxymethyl cellulose (NaCMCXL, AcDiSol^®^SD-711, FMC Europe NV, Brussels, Belgium) and polyvinyl polypyrrolidone (PVPP, Polyplasdone™ XL, Ashland, Schaffhausen, Switzerland) were employed as disintegrants. Rhodamine B (RhB, Merck, Darmstadt, Germany) was used as fluorescent dye. Aqueous dispersions of spherical polyethylene (PE) particles of four different particle size ranges (45–53 µm, 125–150 µm, 355–425 µm, 710–850 µm) containing an orange fluorescent dye (Cospheric LLC, Santa Barbara, CA, USA) were prepared with Polysorbate 80 (Tween^®^ 80, Merck, Darmstadt, Germany) as dispersant and used to verify the analytical method. The dispersion with the finest PE particles was additionally used as calibration suspension. Demineralised water at 22 ± 2 °C with a pH of 7 was used as test medium throughout the study.

### 2.2. Polymer Powder Manufacturing

Both pharmaceutical polymers and 0.1% *w/w* RhB fluorescent dye for selective tracing were blended with a laboratory mixer (Turbula^®^, Willy A. Bachofen AG, Basel, Switzerland) at 34 rpm for 20 min. The blends were processed with a twin-screw extruder (ZE 9 HMI, Three-Tec GmbH, Seon, Switzerland) with a 2 mm rod die. The extrudates were cut manually and milled with a hammer mill (MF 10/10.2, IKA, Staufen, Germany) at 4000 rpm, in which the product passed through an inserted sieve with 500 µm mesh size (MF 0.5, IKA, Staufen, Germany) to obtain powders suitable for tablet compaction. 

### 2.3. Particle Size Analysis

The particle size distributions of the pharmaceutical polymer powders and the spherical PE particles were determined in wet dispersed condition by laser diffraction (LD, HELOS/KR + QUIXEL, Sympatec, Clausthal-Zellerfeld, Germany) according to Fraunhofer theory. n-heptane (n-heptane 99%, Brenntag, Basel, Switzerland) was used as dispersion medium for Eudragit EPO and White Spirit (White Spirit/Terlitol 16/18%, Brenntag, Basel, Switzerland) was used as dispersion medium for Kollidon VA64 and the PE particles.

Additionally, a scanning electron microscope (SEM, GeminiSEM 300, Zeiss, Oberkochen, Germany) was used to allow a visual investigation of size and shape of the pharmaceutical polymer powders and the PE particles.

### 2.4. Tablet Manufacturing

The pharmaceutical polymers and other excipients employed for each formulation were blended with a laboratory mixer (Turbula^®^, Willy A. Bachofen AG, Basel, Switzerland) at 34 rpm for 20 min. The content of the pharmaceutical polymer in the blend was always 20%. Throughout this study, the declared compositions of formulation components are related to their mass. 

A single punch tablet press (Styl’one Evolution Compaction Simulator 578, Medelpharm S.A.S., Beynost, France) was used for the preparation of round flat-faced tablets with a constant tablet mass of about 500 mg. Compressibility profiles were generated for each formulation in a linearly subdivided range between 20 and 300 MPa to obtain different tablet porosities. Throughout all compaction processes, the compaction profile StylCamDirectCam with a compaction speed of 5 rpm was applied using 11.28 mm Euro-D punches and a paddle feed shoe.

The diameter and height of each tablet were measured by a micrometre (IP65, Mitutoyo Schweiz AG, Urdorf, Switzerland) and the mass was determined by an external balance (AT261 DeltaRange^®^, Mettler Toledo Schweiz GmbH, Greifensee, Switzerland) prior to the tablet disintegration analysis. A tablet relaxation period of 7 days at controlled room temperature (22 ± 2 °C) and humidity (35 ± 5% rh) was considered prior to geometry and mass determination.

### 2.5. Tablet Water Uptake and Swelling Analysis

The method for the simultaneous and time-resolved analysis of water uptake and swelling of tablets used in this study was presented and validated in a previous publication [40]. In brief, the experimental set-up was designed to allow water penetration through the tablets’ front face. Water uptake was determined with a balance (PR1203, Mettler Toledo Schweiz GmbH, Greifensee, Switzerland) by measuring the increase in tablet mass and swelling was determined with a digital camera (Dimax HS4, PCO AG, Kelheim, Germany) by measuring the increase in tablet volume. A self-developed algorithm for the symmetry-based 3D volume reconstruction was applied to obtain volumes of the tablets from 2D images.

### 2.6. Standard Tablet Disintegration Analysis

A standard disintegration test (SDT) set-up according to USP 701, Ph.Eur. 2.9.1 or JP 6.09 (DisiTest 50, SOTAX AG, Aesch, Switzerland) was used to determine the DT of the tablets [6,7,8]. The DT was determined for each tablet when complete disintegration had been reached, which is defined as the time when there was no more residue remaining on the screen. Each tablet formulation was tested in triplicate at standard conditions of 30 strokes per minute and modified conditions of 5 strokes per minute, respectively. The resulting average superficial fluid velocities inside the tubes were estimated based on the average basket velocity to be 55 and 9 mm/s, respectively [9].

### 2.7. Novel Tablet Disintegration and Dispersion Analysis

#### 2.7.1. Experimental Set-Up

**Figure 1:**
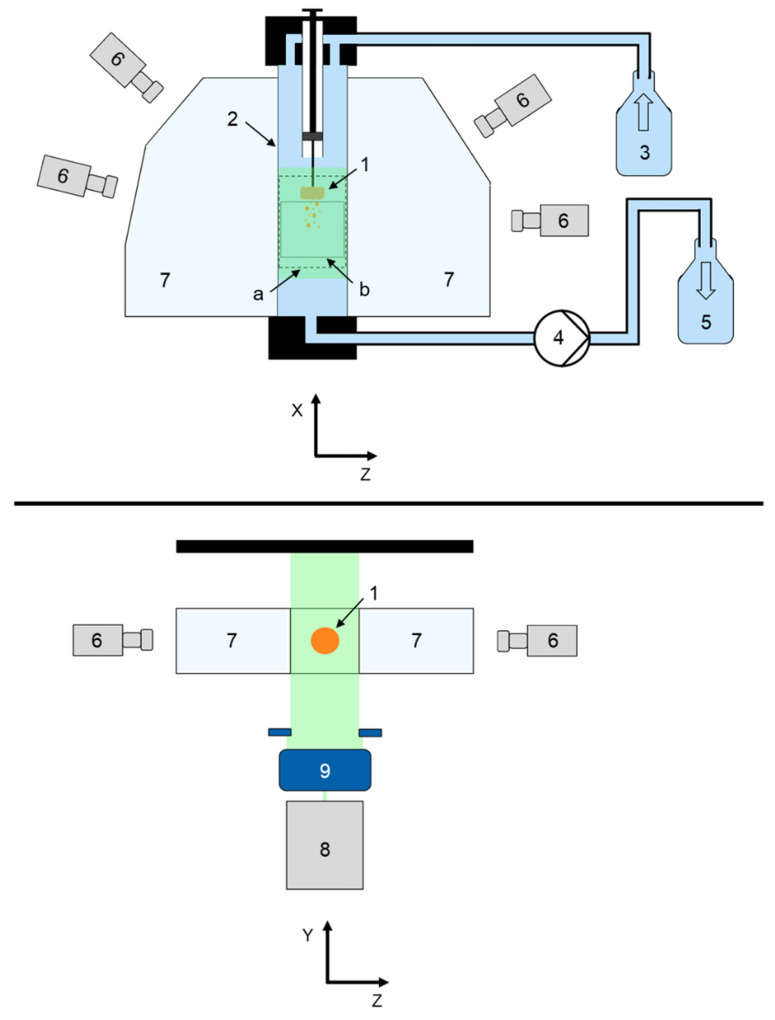
Schematic diagram of the 3D Tomo-LIF experimental set-up in front view (**top**) and top view (**bottom**); 1: tablet, 2: flow channel, 3: water reservoir, 4: peristaltic pump, 5: waste container, 6: digital cameras, 7: water-filled prisms, 8: laser (emitted laser light displayed green), 9: volume optics, (a): measurement volume (dashed line), (b): analysis volume (dotted line).

Three-dimensional tomographic laser-induced fluorescence imaging (3D Tomo-LIF) was examined to investigate the disintegration and dispersion of tablets. Figure 1 shows a schematic diagram of the experimental set-up for the time-resolved analysis to determine tablet DT and particle dispersion after disintegration immediately below the tablet in a vertical flow channel (2 in Figure 1) with a quadratic cross-sectional area of 20 mm × 20 mm and a length of 300 mm.

Demineralised water was pumped from a reservoir (3 in Figure 1) in a top to bottom flow direction through the flow channel in single-pass mode by a peristaltic pump (4 in Figure 1; Masterflex^®^ L/S^®^, Cole-Parmer, Roissy, France) and collected in a waste container (5 in Figure 1). The water reservoir, flow channel and waste container were connected via tubing (T3306-33, Saint Gobain, La Défense, France).

**Figure 2:**
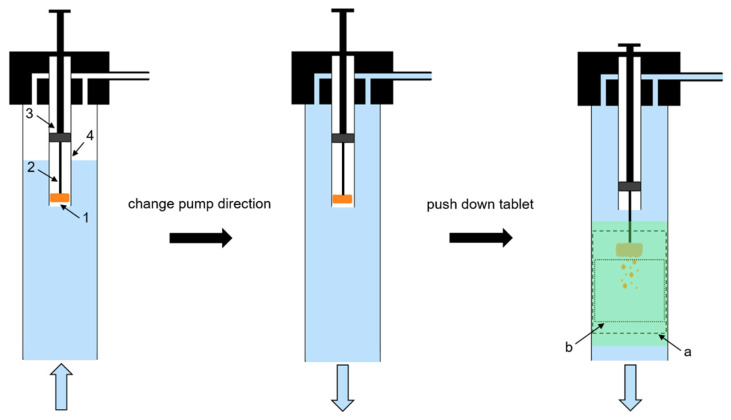
Schematic diagram of the tablet positioning system (TPS) during the filling procedure (**left**), after the change of pump direction (**centre**) and during the measurement procedure (**right**); 1: tablet, 2: wire tablet holder, 3: plunger, 4: cylindrical tube, (a): measurement volume (dashed line), (b): analysis volume (dotted line).

A tablet positioning system (TPS) was constructed to realise a fast and reproducible positioning of the tablet in the steady-state top to bottom flow (Figure 2). The tablet (1 in Figure 1 and Figure 2) was fixed with a bent wire tablet holder (2 in Figure 2), which was mounted at the bottom of a plunger (3 in Figure 2) in a cylindrical tube (4 in Figure 2). When the plunger was in the pulled-up position, tablet and wire were entirely enclosed within the tube. The interface between the bottom of the plunger and the wall of the cylindrical tube was gas-tight. When the plunger was pushed down, tablet and wire protruded out of the tube. The TPS was mounted at the upper opening of the flow channel and connected to the inlet tubing. A 3D Tomo-LIF imaging system (FlowMaster, LaVision GmbH, Göttingen, Germany) was used to record a measurement volume (a in Figure 1 and Figure 2) of 20 mm × 20 mm × 70 mm, which included the tablet, and an analysis volume (b in Figure 1 and Figure 2) of 20 mm × 20 mm × 25 mm located immediately below the tablet. Therefore, within the measurement volume the DT of the tablet was determined and the dispersion analysis of disintegrated particles was performed. The 3D Tomo-LIF imaging system consisted of four digital cameras (6 in Figure 1) arranged in such a way that the lines of sight were perpendicular to the Y-axis and at angles of −75°, −45°, 60° and 90° to the X-axis. Camera lenses with a focal length of 25 mm and an adjusted f-number of 8 were used for each camera. The f-number is the ratio of the focal length to the diameter of the effective aperture.

Tilting of the camera sensor relative to the camera lens allowed a Scheimpflug correction to adjust the focal plane to the XY-plane for all cameras. Water-filled prisms (7 in Figure 1) between the flow channel and the cameras minimised the effect of refraction, as the lines of sight were perpendicular to the interface between air and water. A Nd:YAG SHG laser (8 in Figure 1; NANO-L 145-15, Litron Lasers Ltd., Rugby, UK) with a wavelength of 532 nm and volume optics for beam expansion (9 in Figure 1) realised the illumination of the desired measurement volume. The laser and the cameras were synchronised by a programmable timing unit. The fluorescent dye molecules in the measurement volume were excited by the laser light and emitted light with a wavelength between approximately 530 and 650 nm [41]. A longpass filter was mounted on each camera lens to filter any light below 542 nm, including the emitted light of the laser. Throughout the analysis, the 3D Tomo-LIF imaging system was operated by the software DaVis 10.1. (LaVision GmbH, Göttingen, Germany).

#### 2.7.2. Experimental Procedure

The measurement volume was calibrated by a two-level double-sided 3D calibration plate. Each camera took one image of the plate at three different positions on the Z-axis. Accordingly, mapping functions were calculated for all cameras and stored in DaVis. Prior to each analysis, the TPS was dismantled and the plunger was pushed down to manually place the tablet in the tablet holder. Then, the plunger was pulled up and the TPS was mounted on the flow channel. The entire flow channel and tubing were filled with water by operating the peristaltic pump in reverse direction, whereby the tablet was protected from water contact inside the tube by the air reservoir during the filling procedure (Figure 2, left). Subsequently, the pump direction was changed to set a steady-state top to bottom flow in the channel (Figure 2, centre). The camera recording and the laser emission were initiated at a frame rate and repetition rate, respectively, of 10 Hz. By pushing down the plunger, the tablet was positioned in the flow to simultaneously define the start of the measurement and initiate the monitoring of the entire disintegration and dispersion analysis (Figure 2, right).

#### 2.7.3. Disintegration Time Analysis

The DT was determined at the point when all tablet fragments, which could be agglomerates, aggregates or primary particles, were liberated from the tablet holder. Once this criterion was met, or when the disintegration process exceeded 10 min, the analysis was stopped. The flow channel and tubing were emptied and the calibration suspension was pumped through, whereof a series of 300 images was recorded. Finally, the calibration suspension was removed, the TPS was dismantled and the tablet holder, flow channel and tubing were cleaned. Aqueous dispersions of the PE particles were pumped through the flow channel and recorded for a preceding method verification without using the TPS.

#### 2.7.4. Dispersion Analysis

After each experiment, the particle reconstruction was performed in DaVis. At first, a volume self-calibration algorithm using the images of the calibration suspension was applied to correct the mapping function of all cameras, resulting in an improved calibration accuracy [42,43]. The raw images of the disintegrating tablet were pre-processed by setting a segmentation threshold where all signals with lower intensity were eliminated. The segmentation threshold value was determined by analysing the raw images for each polymer type based on the ratio of maximum intensity to the diameter of particles. A reference value for the segmentation threshold was obtained using the PE particles, which is described in Section 3.3. The increment between the images was adjusted in each analysis according to the vertical particle velocity in order to avoid multiple counting of particles. Then, the area of interest for the volume reconstruction was defined by applying a mask on each perspective, which was directly below the tablet. Finally, the analysis volume was reconstructed from the masked images and the size and position of each particle in this analysis volume at any certain time point of the measurement was obtained. The resulting voxels had a size of approximately 54 µm^3^. Further data processing was performed with a self-written Python software (Anaconda Software Distribution, Version 5.2.0, Anaconda Inc., Austin, TX, USA, https://anaconda.com, accessed on 29 May 2018) to divide the measured particles in certain time intervals into defined particle size classes.

## 3. Results and Discussion

### 3.1. Tablet Water Uptake and Swelling

**Figure 3:**
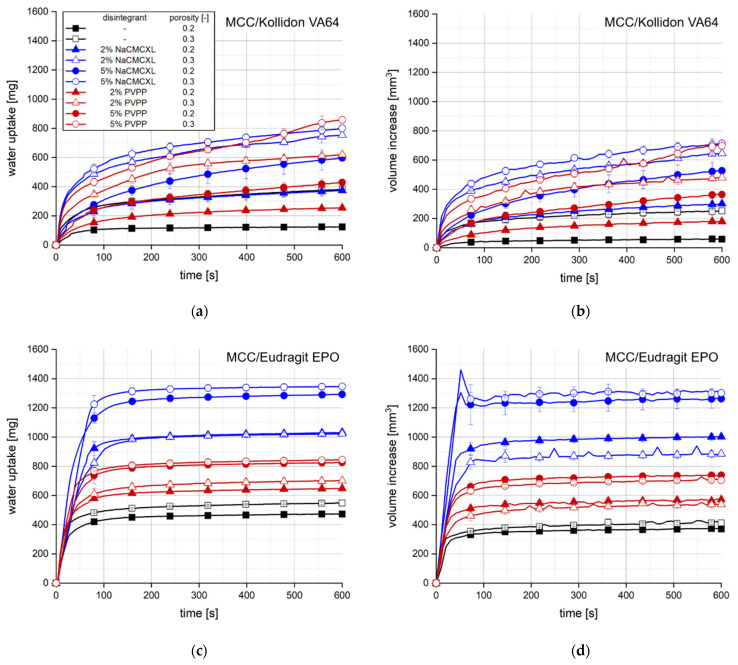
Water uptake (**a**,**c**) and volume increase (**b**,**d**) with standard deviation (*n* = 3) of tablets containing MCC and 20% of either Kollidon VA64 (**a**,**b**) or Eudragit EPO (**c**,**d**). NaCMCXL and PVPP were used as disintegrants; the line represents the connection of 7 data points per second for water uptake and 6 data points per minute for volume increase.

Figure 3 shows the results of the water uptake and swelling analysis of the tablets based on MCC containing 20% Kollidon VA64 (Figure 3a,b) or Eudragit EPO (Figure 3c,d) and optionally, disintegrants. The primary particles of the pharmaceutical polymers in the tablets had a rather angular, slightly elongated shape and a particle size x_50,3_ of about 180–200 µm, which was determined by LD and visually confirmed by SEM imaging (data not shown). 

Tablet porosity showed the most significant impact on water uptake and volume increase for tablets containing Kollidon VA64, as both parameters strongly increased with increasing porosity. Higher disintegrant content also resulted in an increase in water uptake and volume increase. Further, water uptake and volume increase were slightly higher when NaCMCXL was used as disintegrant compared to PVPP. Contrarily, the disintegrant type strongly impacted the water uptake and swelling of tablets containing Eudragit EPO as higher values were measured when NaCMCXL was used compared to PVPP. Moreover, a higher disintegrant content led to noticeably higher water uptake and volume increase. However, higher tablet porosity did not or only slightly increase water uptake. For tablets containing PVPP or 2% NaCMCXL the volume increase was even slightly higher with a tablet porosity of 0.2 compared to 0.3. This might indicate that pore penetration and disintegrant functionality were not influenced by the porosity of these formulations, resulting in a similar outcome after the analysis regarding water content and volume. Swelling of the Eudragit EPO particles was not expected at a pH of 7 [44]. A maximum volume increase followed by a fast decrease to a nearly constant value was observed after 50 s for tablets containing 5% NaCMCXL. The maximum might result from a partially air-filled pore volume generated by the strong volume expansion of the disintegrant until pore penetration was completed throughout the tablet. Once this point was approached, the generated pore structure might have partially collapsed due to gravitation.

In general, a much higher water uptake and volume increase were measured for tablets containing Eudragit EPO compared to those containing Kollidon VA64, although the wettability of Kollidon VA64 is significantly higher compared to Eudragit EPO [45,46]. This might be explained by the faster dissolution kinetics of Kollidon VA64 and the consequential increase in viscosity of the penetrating fluid, leading to the formation of a GPN. Hence, further penetration of the tablet pores as well as the volume expansion of the disintegrants and the hydrophilic filler MCC are hindered [37]. 

### 3.2. Standard Tablet Disintegration Analysis

**Figure 4:**
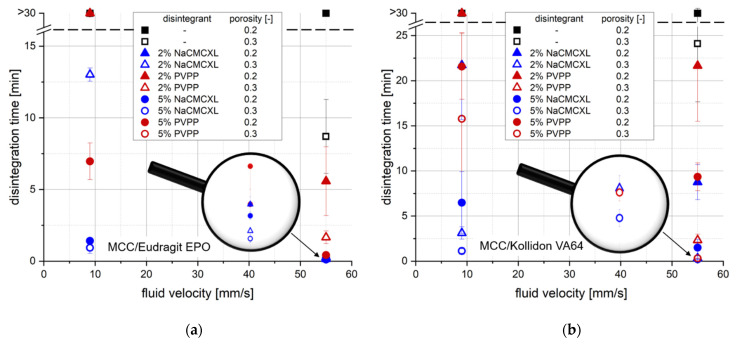
Disintegration time (DT) with standard deviation (*n* = 3) determined by the standard disintegration test (SDT) at standard (55 mm/s) and modified (9 mm/s) average fluid velocity of tablets containing MCC and 20% of either Kollidon VA64 (**a**) or Eudragit EPO (**b**). NaCMCXL and PVPP were used as disintegrants.

Figure 4 shows the DTs of the MCC-based tablets containing Eudragit EPO (Figure 4a) or Kollidon VA64 (Figure 4b) as determined by the SDT at a standard average fluid velocity of 55 mm/s and a modified average fluid velocity of 9 mm/s, which was within the range of average in vivo-like fluid velocities [6,7,8,9,17,21,22,23]. At 55 mm/s all tablets containing Eudragit EPO disintegrated within 1 min when NaCMCXL was used as disintegrant. Tablets with PVPP achieved such fast DTs only with 5% disintegrant content. Moreover, higher tablet porosity resulted in shorter DTs. A noticeable impact of disintegrant content on DT was only observed for PVPP. The shortest DTs for tablets containing Kollidon VA64 were achieved with a porosity of 0.3 at an average fluid velocity of 55 mm/s. In this case, disintegrant type and content only slightly impacted the results. For tablets with a porosity of 0.2, increasing the disintegrant content led to a noticeably faster disintegration. Moreover, NaCMCXL showed a better disintegration behaviour compared to PVPP. At the higher average fluid velocity, only the tablets with a porosity of 0.2 and without disintegrant did not fully disintegrate within 30 min. 

It was not surprising that in general, the DTs strongly increased when the average fluid velocity was reduced from 55 mm/s to 9 mm/s. Only tablets with a porosity of 0.3 and 5% NaCMCXL had completely disintegrated after approximately 1 min. The DTs increased for these formulations containing 5% NaCMCXL when the porosity decreased to 0.2, which was a stronger effect for tablets containing Kollidon VA64 compared to those containing Eudragit EPO. Similarly, when 5% PVPP was used as disintegrant in tablets with Kollidon VA64, the DTs increased with decreasing porosity. In contrast, the DTs of tablets containing Eudragit EPO and 5% PVPP were significantly shorter for a tablet porosity of 0.2 compared to 0.3 at an average fluid velocity 9 mm/s. When the disintegrant content of tablets containing Eudragit EPO was decreased to 2%, only tablets containing NaCMCXL with a porosity of 0.3 completely disintegrated within 30 min and their DTs were much longer compared to the tablets containing 5% NaCMCXL. For tablets containing Kollidon VA64 with a porosity of 0.3, the DTs were only slightly longer with 2% NaCMCXL compared to 5% NaCMCXL. This impact of disintegrant content on the DTs was more pronounced for tablets with a porosity of 0.2. All tablet formulations with 2% PVPP or no disintegrant did not completely disintegrate within 30 min at an average fluid velocity of 9 mm/s.

Table 1 shows a qualitative assessment of the impact of the formulation properties on the DT of the tablets at standard and modified fluid velocity. Each formulation property was categorised individually according to the clarity of the impact on the rank order of the DT. Interestingly, these experiments revealed that the rank order of the DT results changed not only with the polymer type, but also with the average fluid velocity for the tablet formulations under investigation. In particular, the impact of disintegrant content on DT for tablets containing Eudragit EPO, as well as the impact of disintegrant type on DT for tablets containing Kollidon VA64 noticeably increased at modified average fluid velocity. Further, the characteristic behaviour of tablets containing Eudragit EPO and 5% PVPP which disintegrate significantly faster with lower porosity could only be detected at modified average fluid velocity. This might be explained by the commonly accepted property of PVPP to expand by a shape recovery mechanism, which is more pronounced when higher compaction stresses are applied [47,48,49]. During the experiments at standard average fluid velocity, hydrodynamic forces might superimpose this mechanism of disintegration by shape recovery. An explanation for why this effect is not apparent for tablets containing Kollidon VA64 might be that water uptake and, therefore, swelling is impeded by the formation of a GPN, which is more pronounced for tablets with a porosity of 0.2 compared to a porosity of 0.3. In contrast, water uptake and swelling of tablets containing Eudragit EPO is nearly unaffected by porosity (see Section 3.1). 

**Figure 5:**
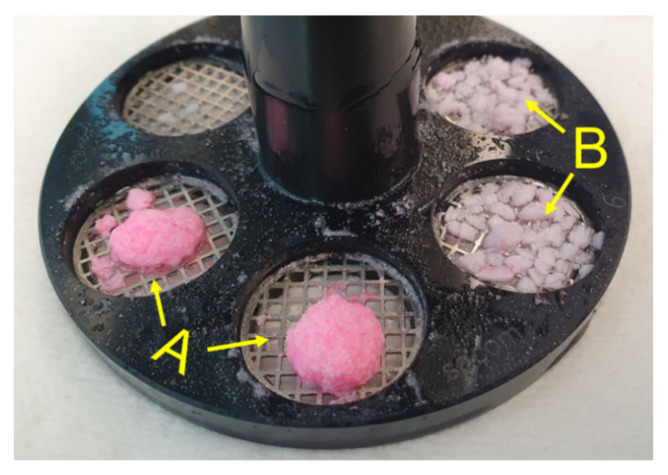
Tablet residues on the screen after a 30 min standard disintegration test (SDT) at modified average fluid velocity (9 mm/s). Coarse tablet residues visible for tablets containing 78% MCC and 20% Kollidon VA64 (A); finer tablet fragments visible for tablets containing 78% MCC and 20% Eudragit EPO (B). All tablets contained 2% PVPP as disintegrant and had a porosity of 0.2.

In general, some characteristics of tablet disintegration could not be distinguished by the SDT due to the criterion of complete disintegration, which is defined as that time when no residue of the tablets was remaining on the screen. Figure 5 shows an example of two tablet formulations, MCC/Kollidon VA64 (A) and MCC/Eudragit EPO (B), each with 2% PVPP. The DTs were >30 min in both cases. However, the disintegration behaviour of the formulations was visibly different. For MCC/Kollidon VA64, disintegration mainly proceeded from the tablet surface and a tablet core was still present after the analysis, whereas MCC/Eudragit EPO tablets fully disintegrated into fragments of a few millimetres in size. Moreover, throughout the analysis of tablets containing Kollidon VA64, partial clogging of the screen by the formed GPN was observed, which was more pronounced at 9 mm/s average fluid velocity.

DT analysis by the SDT required not only the break-up of the tablet structure into fragments, but also a certain dispersion of these fragments to pass finally through the screen to fulfil the disintegration criterion. In such an experiment, disintegration and dispersion could be provoked by both volume expansion or dissolution of the excipients upon water contact and hydrodynamic forces. The high impact of the hydrodynamic forces could be demonstrated by the variation of the average fluid velocity in the SDT. This might explain that disintegrant content seemed to be a less important parameter regarding the DT of tablets containing Eudragit EPO and NaCMCXL at 55 mm/s. The hydrodynamic forces might not have decisively contributed to disintegration and dispersion at modified average fluid velocity. However, DT strongly decreased with increasing disintegrant content, which might be explained by the generation of finer particles due to an increasing number of points of fracture [25]. 

The test results clearly revealed differences regarding the formulation properties and the applied average fluid velocity. However, a thorough understanding of disintegration and dispersion required more information than only the DT. The tablet characterisation by DT derived from SDT is neither time-resolved nor provides information on the dispersion of agglomerates, aggregates and primary particles. Moreover, the partial clogging of the screen by the GPN obviously hindered particles to pass and the resulting DT was increased, although there were no larger remaining tablet fragments. The obtained results highlight the significant influence of the SDT method on DT results, even when operated at modified average fluid velocity. Thus, the DT results are quite method-specific and the informative value for tablet formulation development and biorelevance are questionable, at least for the formulations and conditions under investigation here. 

Consequently, a meaningful disintegration test method should cover the range of in vivo-like hydrodynamic conditions, provide specific size information on the particles liberated upon disintegration and should not use a particle size-based classification barrier, such as a screen, as criterion to determine the DT. 

### 3.3. Verification of Novel Method for Tablet Disintegration and Dispersion Analysis

**Figure 6:**
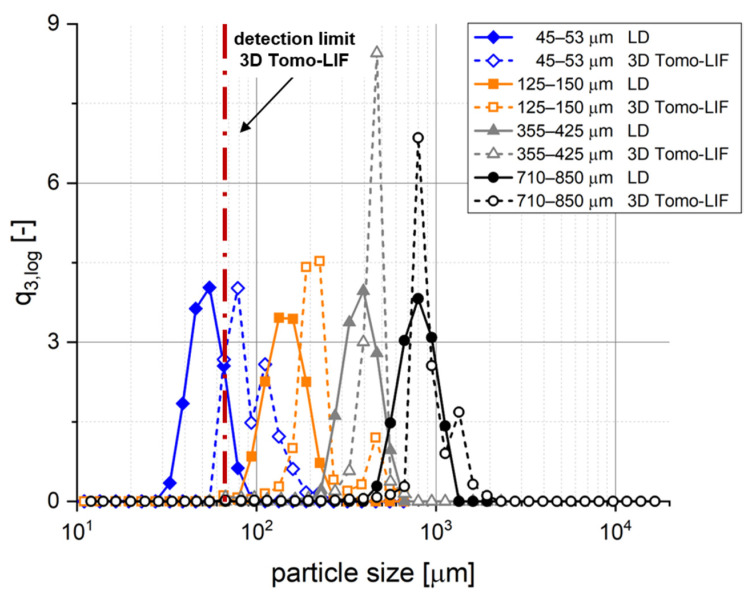
Comparison of particle size distributions (q_3,log_) of the four size ranges of spherical polyethylene (PE) particles determined by laser diffraction (LD) and 3D Tomo-LIF.

The 3D Tomo-LIF method was examined by using dispersed spherical PE particles with four different narrow particle size distributions, representing simple analytical targets regarding their shape for size determination. A high sphericity and a narrow particle size distribution was qualitatively confirmed by SEM imaging (data not shown). Figure 6 and Table 2 show the volume-based particle size distributions and their characteristic values of the spherical PE particles as determined by LD and 3D Tomo-LIF, respectively. In general, the particle sizes determined by 3D Tomo-LIF were higher and had a narrower distribution compared to those determined by LD. The difference in particle size was rather small for the two highest particle sizes of 355–425 µm and 710–850 µm, where the x_50,3_ values of LD and 3D Tomo-LIF did not differ by more than 20%. Contrarily, the x_50,3_ values for the particle size of 45–53 µm and 125–150 µm were about 80% and 43% higher for 3D Tomo-LIF, respectively. This might be explained by the limited resolution of the cameras. It is obvious that 3D Tomo-LIF could not discriminate between particles that were smaller than the voxels. This can be transferred to an equivalent spherical particle diameter of approximately 67 µm defining the detection limit of the method. Further, a bimodal size distribution was determined for the particles with 125–150 µm particle size by 3D Tomo-LIF. The second and smaller peak might be caused by particle agglomeration, which could also be observed on the recorded images, leading to a noticeable increase in the x_50,3_ and x_90,3_. In general, the x_50,3_ values determined by 3D Tomo-LIF were larger than those determined by LD and outside of the range given by the manufacturer. This might be explained by a systematic overestimation of the particle size due to intensity-based particle enlargement artefacts. Although certain differences regarding the resulting particle size distributions were observed between the two analytical methods, the 3D Tomo-LIF method was considered to provide reliable particle size information in the case of particles being coarser than the detection limit of approximately 67 µm. The majority of tablet fragments observable immediately after disintegration were expected to fulfil this criterion in this study, according to the x_50,3_ of the pharmaceutical polymer powders of about 180–200 µm.

### 3.4. Application of Novel Method for Tablet Disintegration and Dispersion Analysis

#### 3.4.1. Investigation of Fluid Velocity

**Figure 7:**
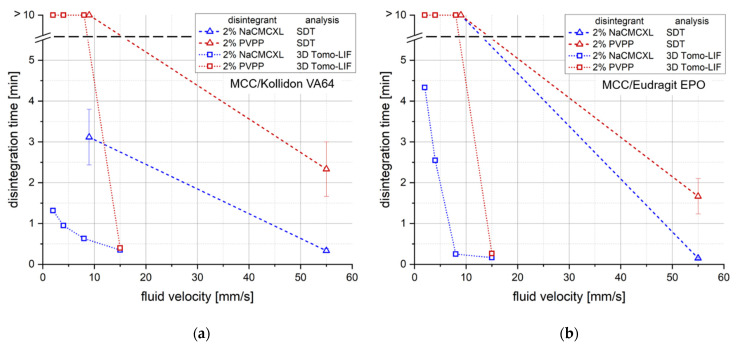
Comparison of disintegration times (DTs) determined by the standard disintegration test (SDT) (*n* = 3) and 3D Tomo-LIF (*n* = 1) with different average fluid velocities for tablets containing 78% MCC and 20% of either Kollidon VA64 (**a**) or Eudragit EPO (**b**). 2% NaCMCXL or PVPP were used as disintegrants and the tablet porosity was 0.3.

The analysis by 3D Tomo-LIF was carried out at superficial fluid velocities between 2 and 15 mm/s to cover the expected range of in vivo-like conditions. Figure 7 displays a comparison of DTs determined by the SDT and 3D Tomo-LIF at varying fluid velocities.

**Figure 8:**
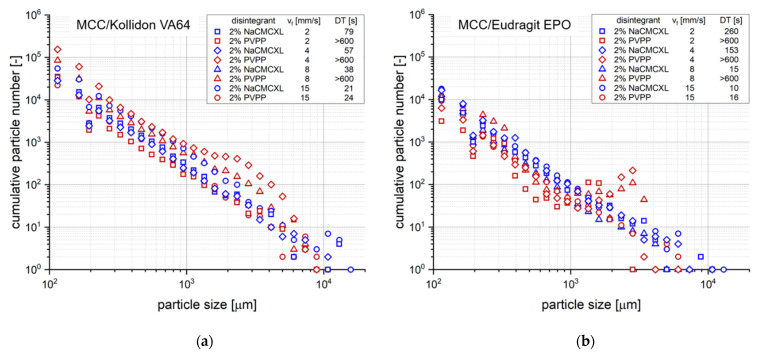
Cumulative number distribution of particles liberated upon disintegration for different average fluid velocities (v_f_) after complete disintegration (refer to DT, respectively) or 10 min of disintegration, respectively, as determined by 3D Tomo-LIF. The tablets contained MCC and 20% of either Kollidon VA64 (**a**) or Eudragit EPO (**b**). 2% NaCMCXL or PVPP was used as disintegrant and the tablet porosity was 0.3.

Additionally, the cumulative particle number for defined particle size classes at DT determined during the disintegration and dispersion analysis by 3D Tomo-LIF is shown in Figure 8. The SDT results at standard fluid velocity of tablets containing 2% of NaCMCXL or PVPP enable an accurate assessment of the formulations on the basis of DTs < 3 min. At modified fluid velocities of SDT, DTs were significantly longer than 10 min except for the formulation with Kollidon VA64 and NaCMCXL. Therefore, the information provided by the SDT at such low fluid velocities is insufficient for an assessment of the tablet disintegration behaviour at biorelevant conditions. The DTs determined by 3D Tomo-LIF, however, seemed to be significantly shorter compared to the SDT at reduced fluid velocities. This might be explained by the experimental set-up of the 3D Tomo-LIF, where agglomerates, aggregates or primary particles of all sizes were able to freely liberate from the tablet surface. In addition, the point of complete disintegration could be determined easily. Thus, an improved investigation of the DT and related dispersion was possible. 

The use of PVPP resulted in a strong increase in DT for all tablets at a fluid velocity of 8 mm/s or lower, where no complete disintegration within 10 min could be observed. In contrast, complete disintegration of all tablets containing NaCMCXL was observed within 5 min, even at the lowest fluid velocity. However, a noticeable increase in DT was also observed with decreasing fluid velocity, which was more pronounced for tablets containing Eudragit EPO compared to those containing Kollidon VA64. This might be explained by the different mechanisms of action of the disintegrants. The volume expansion induced by NaCMCXL is known to be omnidirectional, whereas that by PVPP is unidirectional only [48,49,50]. In case of PVPP, the hydrodynamic forces might have noticeably contributed to the break-up of the tablet, whereas in case of NaCMCXL these forces probably mainly helped to disperse the particles in the flow. Further, it was noticed that DTs increased much more strongly below 8 mm/s for tablets containing Eudragit EPO compared to Kollidon VA64. This might indicate that the hydrodynamic forces strongly dominate the DT of a formulation containing a polymer with a rather low interaction with demineralised water showing a fast water uptake and volume increase behaviour, such as Eudragit EPO (Figure 3c,d). In contrast, disintegration might be facilitated for formulations with highly hydrophilic polymers, such as Kollidon VA64, due to simultaneous processes of dissolution, solubilisation, and disintegrant action. Here, the impact of the hydrodynamic forces is very low. However, the water uptake and volume increase, and hence disintegration might be hindered to a certain extent due to GPN formation. 

In general, according to Figure 8, the results of the particle size analysis upon tablet dispersion showed a high number of fine particles around 100 µm and a continuously decreasing number of particles with increasing size. There were only a few individual particles with a size of approximately 10,000 µm. The detection of these coarse particles was enabled by the close proximity of the analysis volume in the flow direction immediately below the tablet. Further, the extraordinary sizes might be explained by the strong volume expansion of certain tablet excipients, which were present in agglomerates or aggregates mostly towards the end of disintegration. Besides the volume expansion of the solid excipient particles by water absorption, such agglomerates or aggregates were detected as single objects. An extensive liberation of particles within a short time period results in a high local concentration, which may not be discriminated by the measurement system. Therefore, very coarse particles were detected and the cumulative particle volume might be coarser than the actual liberated agglomerate or aggregate particle size. In addition to the resulting particle size information a visual analysis of the raw images might be considered for a better interpretation of the data. At the end of the disintegration process, the break-up of the tablet core into very few but coarse fragments might also be detected. The fixation of the tablet only with a bent wire may promote the disintegration into very few, coarse fragments at the end of the analysis. This might lead to a higher variability of the results at the end of the analysis.

However, the advantage of the 3D Tomo-LIF analysis is the quantitative monitoring of disintegration and dispersion under defined hydrodynamic conditions, aiming for the assessment of the biorelevant inherent functional properties of the tablet. It should be noted that the particle velocities were partially dependent on particle size and position. In general, the velocity decreased with particle size and increasing distance from the centre of the flow channel. The increment between the images for reconstruction was selected based on the fastest particle velocities. Thus, a certain overestimation of the particle number, of the finer particles in particular, must be considered for the interpretation of the data. Apparently, an increased number of particles between approximately 1000 and 5000 µm was observed for tablets containing PVPP at fluid velocities of 4 and 8 mm/s (Figure 8). Such a distinct impact of fluid velocity on the particle size could not be seen for tablets containing NaCMCXL. A possible explanation for this behaviour might be the omnidirectional volume expansion of NaCMCXL, leading to higher dispersion and thus finer particles in that specific range compared to PVPP. Although the tablets with PVPP did not fully disintegrate within 10 min at fluid velocities ≤ 8 mm/s, a high number of particles was still detected for each size class. This might indicate a higher efficacy of disintegration for those tablets than expected solely from comparing DTs, underlining the importance of particle number and size analysis upon disintegration. 

**Figure 9:**
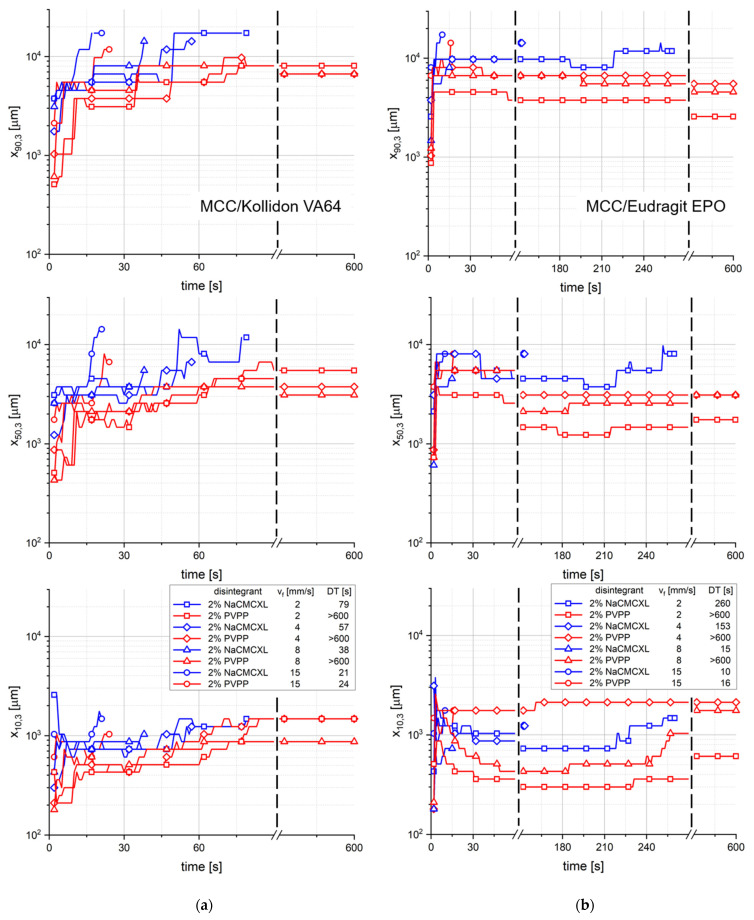
The characteristic particle sizes x_10,3_, x_50,3_ and x_90,3_ of the size distribution of cumulated particles liberated upon disintegration for different average fluid velocities (v_f_) as a function of time, as determined by 3D Tomo-LIF. The tablets contained MCC and 20% of either Kollidon VA64 (**a**) or Eudragit EPO (**b**). 2% NaCMCXL or PVPP was used as disintegrant and the tablet porosity was 0.3.

The characteristic particle sizes x_10,3_, x_50,3_ and x_90,3_ of the size distribution of cumulated particles liberated upon disintegration for different average fluid velocities as a function of time is displayed in Figure 9. x_10,3_, x_50,3_ and x_90,3_ of the formulations containing Kollidon VA64 seemed to increase continuously within the first 90 s of the measurement. Towards the end of the measurements of the tablets with a DT > 600 s, the particle sizes remained constant or decreased slightly. In contrast, for tablets containing Eudragit EPO the x_10,3_, x_50,3_ and x_90,3_ values strongly increased within a few seconds. In the subsequent period of approximately 180 s, the x_10,3_ and x_50,3_ slightly decreased, whereas the x_90,3_ remained nearly constant. Towards the end of the measurements of tablets with a DT > 180 s, the x_10,3_ and x_50,3_ slightly increased again. The results show a major difference between the two polymers regarding the tablet disintegration and dispersion behaviour. Tablets containing Kollidon VA64 rather disintegrate by surface erosion due to the slow water uptake and volume increase kinetics (Figure 3a,b). Therefore, it might be concluded that the deeper the water penetrates the tablet towards its core, the coarser the liberated particles are. Particles being liberated from tablets containing Eudragit EPO seemed to be of a similar size over a long period of the measurement. At the end of the measurements, the liberation of larger fragments from the tablet core resulted in an increase in the x_10,3_, x_50,3_ and x_90,3_. A characteristic impact of the fluid velocity on the evolution of size distribution of the liberated particles could not be detected.

#### 3.4.2. Investigation of Tablet Formulation

**Figure 10:**
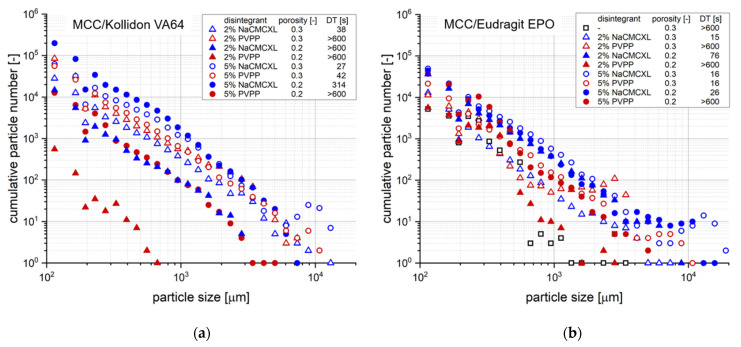
Cumulative number distribution of particles liberated upon disintegration at an average fluid velocity of 8 mm/s after complete disintegration (refer to DT, respectively) or 10 min of disintegration, respectively, as determined by 3D Tomo-LIF. The tablets contained MCC and 20% of either Kollidon VA64 (**a**) or Eudragit EPO (**b**) and had varying tablet porosity, disintegrant type and content. For tablets containing Kollidon VA64 without disintegrant and Eudragit EPO without disintegrant at a porosity of 0.2, no particles were detected.

Figure 10 shows the cumulative particle number distribution after complete disintegration or a 10 min analysis period, respectively, for tablets with varying formulation properties at a fluid velocity of 8 mm/s. An increased number of particles around 10,000 µm could be noticed for tablets with a porosity of 0.3 containing Kollidon VA64 and 5% disintegrant, which might result from a fast and extensive volume expansion due to the high disintegrant content. The tablets fully disintegrated in less than 1 min and the amount of coarse particles indicate the very limited dispersion directly after disintegration. 

In general, both tablet disintegration and dispersion into fine particles are required to increase the API release. On the one hand, a short DT of a tablet does not necessarily imply an adequate dispersion into fine particles. On the other hand, the dispersion behaviour of certain tablets might be underestimated when long DTs are determined by the SDT according to the pharmacopoeial definition of complete disintegration. Therefore, the determination of both the DT and particle sizes after disintegration could provide complementary information to increase the understanding and relationship of both aspects of the tablet performance under controllable and more biorelevant hydrodynamic conditions, compared to the SDT. 

For tablets containing 2% PVPP, full disintegration was not achieved within 10 min. However, the number and size of particles <8000 µm was even slightly higher compared to tablets containing 2% NaCMCXL. Thus, coarse particles of about 10,000 µm might be generated upon break-up of the tablet core into a few fragments only towards the end of the disintegration test, which were not detected for tablets with a DT > 10 min. Complementary to the DT, the assessment of dispersion by particle size analysis in the discrete volume immediately below the tablet operated with continuous flow could further provide information on the mechanism of disintegration, such as break-up of the tablet core or surface erosion. However, there was no example of complete disintegration by surface erosion in this study. 

Decreasing the porosity to 0.2 induced a strong increase in DT for all formulations containing Kollidon VA64. Full disintegration was achieved only when 5% NaCMCXL was used and even a slightly higher number of particles ≤5000 µm compared to the tablets with higher porosity was measured. In contrast, a noticeably lower number of particles was observed for tablets containing PVPP or 2% NaCMCXL, which was especially pronounced when 2% PVPP was used. Moreover, the coarsest detected particles were finer compared to the tablets with a porosity of 0.3. When 2% PVPP was used, the coarsest particles were <700 µm in size. This might be closely related to the low water uptake and volume increase measured for these tablets, indicating hindered water penetration due to the formation of a GPN (Figure 3a,b). Thus, disintegration might have occurred only at the tablet surface, leading to the reduced number and size of particles formed [51]. At lower porosity, the disintegration performance of NaCMCXL seemed to be advantageous compared to PVPP. No liberation of particles was detected throughout the analyses of tablets containing Kollidon VA64 without disintegrant. 

For formulations with Eudragit EPO and 5% disintegrant, an increased number of coarse particles liberated from tablets with a porosity of 0.3 compared to those with a porosity of 0.2. It might be concluded that a high disintegrant content led to strong volume expansion within the tablet and that the very coarse particles were likely to be agglomerates or aggregates of the two pharmaceutical polymers and other excipients. Thus, the method might be tainted with an uncertainty regarding the selective detection of pharmaceutical polymers. With this consideration, the selective detection might be limited to primary particles, whereas in case of agglomerated or aggregated particles the whole objects including other excipients were measured. 

Except for tablets with 5% disintegrant and a porosity of 0.3, DT was significantly shorter when NaCMCXL was used compared to PVPP. Moreover, the number of liberated particles of nearly every size class was higher for tablets containing NaCMCXL compared to those containing PVPP with comparable disintegrant content and porosity. Despite the relatively high water uptake and volume increase, the combination of disintegrant action of PVPP and hydrodynamic forces at a fluid velocity of 8 mm/s for 10 min was not sufficient to induce a full disintegration of the tablets. The lowest number of particles for nearly all particle size classes was measured for the tablet containing 2% PVPP with a porosity of 0.2 and the tablet without disintegrant with a porosity of 0.3. This was particularly apparent for particles > 1000 µm, where only very few particles were detected. No detachment of particles was detected throughout the analysis of the tablet containing Eudragit EPO with a porosity of 0.2 without disintegrant. In general, NaCMCXL might be considered as the disintegrant with the higher efficacy, according to the results. 

**Figure 11:**
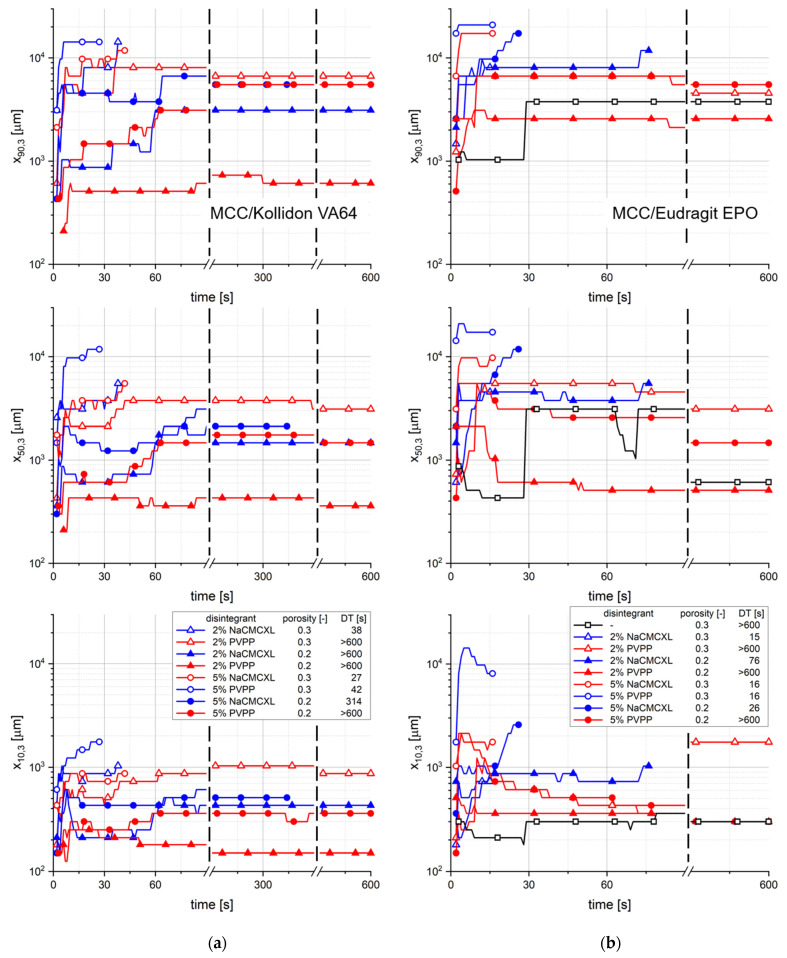
The characteristic particle sizes x_10,3_, x_50,3_ and x_90,3_ of the size distribution of cumulated particles at an average fluid velocity of 8 mm/s as a function of time, as determined by 3D Tomo-LIF. The tablets contained MCC and 20% of either Kollidon VA64 (**a**) or Eudragit EPO (**b**) and had varying tablet porosity, disintegrant type and content. For tablets containing Kollidon VA64 without disintegrant and Eudragit EPO without disintegrant at a porosity of 0.2, no particles were detected.

The characteristic particle sizes x_10,3_, x_50,3_ and x_90,3_ of the cumulative size distribution of particles liberated upon disintegration at an average fluid velocity of 8 mm/s as a function of time is displayed in Figure 11. For most of the tablet formulations containing Kollidon VA64, the x_50,3_ and x_90,3_ continuously increase within the first 90 s. In the further course of the measurement, the particle size distribution remained nearly constant. For tablets containing Eudragit EPO, the characteristic particle sizes strongly increased within a few seconds after the start of the measurement. In the further course, the values remained constant or slightly decreased, which was more pronounced for the x_10,3_ and x_50,3_. No distinct impact of porosity, disintegrant type or content on the evolution of the particle size distribution could be observed. The results might underline the hypothesis that in the initial phase increasingly coarser particles liberated from Kollidon VA64-based formulations probably due to the disintegration by surface erosion.

**Figure 12:**
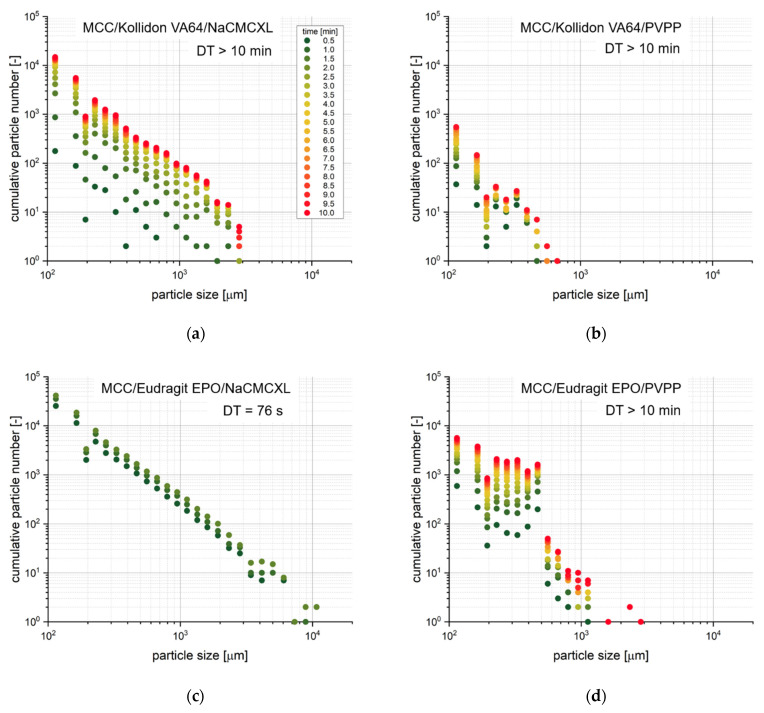
Time-resolved cumulative number distributions of liberated particles determined by 3D Tomo-LIF at an average fluid velocity of 8 mm/s. The tablets contained 78% MCC, 20% of either Kollidon VA64 (**a**,**b**) or Eudragit EPO (**c**,**d**) and 2% of either NaCMCXL (**a**,**c**) or PVPP (**b**,**d**). The tablet porosity was 0.2.

Figure 12 provides time-resolved cumulative size distributions on the dispersion of selected tablets with 2% disintegrant content and a porosity of 0.2. The MCC/Eudragit EPO tablet containing NaCMCXL fully disintegrated within 2 min and a majority of the particles of each size class ≤6000 µm was measured already after 30 s. All other tablets did not completely disintegrate. However, differences in particle size, number and time point of disintegration could be observed. For the MCC/Kollidon VA64 tablet containing NaCMCXL, a large number of particles were formed already within 4 min, whereas only a few particles were liberated between 4 and 8 min. All particles were finer than 3000 µm. When PVPP was used as disintegrant instead, a large change in the number of particles was measured between 5 and 10 min. No particles in the size classes above 500 µm were observed for PVPP in MCC/Eudragit EPO or MCC/Kollidon VA64 formulation. By far the lowest number of particles was detected for the MCC/Kollidon VA64 tablet. In general, it could be observed that the coarsest particles were liberated rather at a later stage of the analysis. This might indicate that only finer particles liberated from the tablet surface region at the beginning, which could be described as surface erosion due to the probably incomplete wetting of the tablet core. In the further course of the analysis, the volume expansion of the disintegrant particles within the wet but stable tablet core was not sufficient to induce complete disintegration at low fluid velocity. However, a few individual coarser agglomerates or aggregates were liberated more or less steadily. The systematically decreased particle number in the size class at 194 µm may be described as a statistical artefact due to the close proximity to the detection limit of the 3D Tomo-LIF system.

Table 3 shows a comparison of the DTs determined by SDT at modified (9 mm/s) average fluid velocity and 3D Tomo-LIF at an average fluid velocity of 8 mm/s for different tablet formulations. According to the estimated average fluid velocity in both experiments, an approximate comparability regarding the hydrodynamic conditions for the different methods is assumed. However, it must be considered that the flow patterns and the minimum and maximum fluid velocities are noticeably different. For half of the investigated formulations, DT exceeds 10 min in both the SDT and 3D Tomo-LIF experiment. When 5% NaCMCXL was used, the differences between the DTs determined by SDT and 3D Tomo-LIF for each formulation were less than 1 min. In contrast, for the tablet formulations containing 5% PVPP with a porosity of 0.3 significantly shorter DTs were determined by 3D Tomo-LIF compared to the SDT. The different results can be explained by the method-specific definitions of complete disintegration. The DT as determined by SDT expresses both the disintegration and a certain state of particle dispersion due to the relatively small mesh size of the screen (Figure 5). Therefore, the SDT does not provide a discriminative information between the processes of disintegration and dispersion, but a rather method-specific result of limited value in relation to the in vivo behaviour. In contrast, DT determined by 3D Tomo-LIF was defined when all fragments were liberated from the tablet holder, regardless of their size. Consequently, the process of disintegration was investigated separately from the process of dispersion. The experiment results of 3D Tomo-LIF, DT in combination with time-resolved particle size information, provide a more comprehensive understanding of the two processes and revealed that the tablets seemed to disintegrate relatively fast, but the dispersion of the liberated fragments was limited. Only in the experiments with tablets containing Eudragit EPO and 5% PVPP with a porosity of 0.2 was the DT determined by SDT shorter compared to the DT determined by 3D Tomo-LIF. This exceptional behaviour might be explained by a possible limitation of the axial shape recovery mechanism of PVPP in the tablets by the tablet holder used for the 3D Tomo-LIF experiments.

## 4. Conclusions

In this work, a device was designed to study the disintegration and dispersion of tablets containing pharmaceutical polymers under in vivo-like hydrodynamic conditions. The tablets were positioned in a continuously operated flow channel in single-pass mode with a flow direction from top to bottom in order to simulate low to medium fluid velocities. DT was determined and time-resolved size information on the particles formed upon disintegration was obtained in a discrete analysis volume immediately below the tablet by a novel 3D Tomo-LIF method. The experimental results were compared with results obtained from the SDT at an estimated average fluid velocity of 55 mm/s (standard) and of 9 mm/s (modified), based on the sinusoidal basket velocity.

The SDT results provide insufficient information to fully understand the disintegration and dispersion behaviour of tablets, as both processes could not be discriminated. A comparison between standard and modified fluid velocity shows a change of the rank order of DT for some formulations. Hydrodynamic forces substantially contribute to disintegration and dispersion at standard fluid velocity and potentially superimpose or significantly alter the functional behaviour of the tablet, which provokes a misleading interpretation of the in vivo performance. The analysis by 3D Tomo-LIF provides complementary and in-depth information on tablet disintegration and dispersion under defined and adjustable hydrodynamic conditions in the range of in vivo-like fluid velocities occurring most often in the human stomach in the fasted and fed states. The analytical capability and precision of the 3D Tomo-LIF method was demonstrated for tablets containing pharmaceutical polymers used for ASDs labelled with a fluorescent dye under steady state hydrodynamic conditions with fluid velocities between 2 and 15 mm/s. Differences in disintegration behaviour in dependence on polymers, disintegrants, formulation compositions and tablet porosities could be successfully characterised and discriminated. A more detailed understanding of the functional behaviour of tablet material, composition and structural properties under in vivo-like hydrodynamic forces could be gained through the complementary results of DT and particle size obtained by 3D Tomo-LIF. Thus, we consider the 3D Tomo-LIF in vitro method to be of improved biorelevance in terms of hydrodynamic conditions in the human stomach in the fasted and fed states.

For future work, the 3D Tomo-LIF method could be combined with a particle tracking algorithm to mitigate possible uncertainties regarding the particle number due to varying particle velocities. Further, the system might be adapted to analyse disintegration and dispersion using biorelevant test media at body temperature. A combination of disintegration, dispersion and dissolution analysis could be enabled by integration of an inline UV/Vis spectrometer in the set-up connected to the outlet of the flow channel. In addition, the method could be further developed to a simplified 3D tomographic imaging method analysing the entire particulate material during dispersion, thus enabling the general applicability for any solid oral dosage form.

## Figures and Tables

**Table 1:** Qualitative assessment of the impact of formulation properties on DT determined by the SDT at standard and modified average fluid velocity; +++: high impact, ++: moderate impact, +: low impact.

Formulation	MCC/Eudragit EPO			MCC/Kollidon VA64		
average fluid velocity	disintegrant type	disintegrant content	tablet porosity	disintegrant type	disintegrant content	tablet porosity
standard 55 mm/s	+++	+	+	+	+	++
modified 9 mm/s	++	+++	++	+++	++	++

**Table 2:** Particle size x_10,3_, x_50,3_ and x_90,3_ of the spherical PE particles, determined by laser diffraction (LD) and 3D Tomo-LIF for each size range.

PE Particles Nominal Size Range (µm)	x_10,3_ (µm)		x_50,3_ (µm)		x_90,3_ (µm)	
	LD	3D Tomo-LIF	LD	3D Tomo-LIF	LD	3D Tomo-LIF
45–53	40	65	52	94	69	136
125–150	106	168	145	207	198	463
355–425	283	359	380	446	500	490
710–850	579	871	791	957	1048	1313

**Table 3:** Comparison of DTs determined by SDT at modified (9 mm/s) average fluid velocity and 3D Tomo-LIF at an average fluid velocity of 8 mm/s for different tablet formulations. The tablets contained MCC and 20% of either Kollidon VA64 or Eudragit EPO and had varying tablet porosity, disintegrant type and content.

Formulation	Composition (% *w/w*)	Porosity (-)	DT (s)	
			SDT	3D Tomo-LIF
MCC/Kollidon VA64	80/20	0.3	>600	>600
MCC/Kollidon VA64	80/20	0.2	>600	>600
MCC/Kollidon VA64/NaCMCXL	78/20/2	0.3	187	38
MCC/Kollidon VA64/PVPP	78/20/2	0.3	>600	>600
MCC/Kollidon VA64/NaCMCXL	78/20/2	0.2	>600	>600
MCC/Kollidon VA64/PVPP	78/20/2	0.2	>600	>600
MCC/Kollidon VA64/NaCMCXL	75/20/5	0.3	68	27
MCC/Kollidon VA64/PVPP	75/20/5	0.3	>600	42
MCC/Kollidon VA64/NaCMCXL	75/20/5	0.2	389	314
MCC/Kollidon VA64/PVPP	75/20/5	0.2	>600	>600
MCC/Eudragit EPO	80/20	0.3	>600	>600
MCC/Eudragit EPO	80/20	0.2	>600	>600
MCC/Eudragit EPO/NaCMCXL	78/20/2	0.3	>600	15
MCC/Eudragit EPO/PVPP	78/20/2	0.3	>600	>600
MCC/Eudragit EPO/NaCMCXL	78/20/2	0.2	>600	76
MCC/Eudragit EPO/PVPP	78/20/2	0.2	>600	>600
MCC/Eudragit EPO/NaCMCXL	75/20/5	0.3	56	16
MCC/Eudragit EPO/PVPP	75/20/5	0.3	>600	16
MCC/Eudragit EPO/NaCMCXL	75/20/5	0.2	85	26
MCC/Eudragit EPO/PVPP	75/20/5	0.2	418	>600

## Data Availability

Data sharing is not applicable to this article.
